# Enhance the Performance of Expectation Propagation Detection in Spatially Correlated Massive MIMO Channels via DFT Precoding

**DOI:** 10.3390/e27101030

**Published:** 2025-10-01

**Authors:** Huaicheng Luo, Jia Tang, Zeliang Ou, Yitong Liu, Hongwen Yang

**Affiliations:** 1 School of Future, Beijing University of Posts and Telecommunicaitons, Beijing 100876, China; luohuaicheng@bupt.edu.cn (H.L.); tangjia@bupt.edu.cn (J.T.); 2 School of Information and Communication Engineering, Beijing University of Posts and Telecommunicaitons, Beijing 100876, China; ouzeliang@bupt.edu.cn (Z.O.); yanghong@bupt.edu.cn (H.Y.)

**Keywords:** massive MIMO, expectation propagation, spatial correlation, precoding

## Abstract

Expectation Propagation (EP) has emerged as a promising detection algorithm for large-scale multiple-input multiple-output (MIMO) systems owing to its excellent performance and practical complexity. However, transmit antenna correlation significantly degrades the performance of EP detection, especially when the number of transmit and receive antennas is equal and high-order modulation is adopted. Based on the fact that the eigenvector matrix of the channel transmit correlation matrix approaches asymptotically to a discrete Fourier transform (DFT) matrix, a DFT precoder is proposed to effectively eliminate transmit antenna correlation. Simulation results demonstrate that for high-order, high-dimensional massive MIMO systems with strong transmit antenna correlation, employing the proposed DFT precoding can significantly accelerate the convergence of the EP algorithm and reduce the detection error rate.

## 1. Introduction

With the rapid evolution of 5G and the advent of 6G, demands for spectral and energy efficiency have spurred interest in massive multiple-input multiple-output (mMIMO) technology [1,2,3]. In a rich scattering wireless propagation environment, equipping transmitters and receivers with a larger number of antennas will increase the degree of freedom (DoF) of the channel and hence the multiplexing gain. Consequently, the system spectral efficiency can be considerably increased. On the other hand, the signals received by the receiver through multiple antennas are a linear mixture of a large number of transmitted symbols with noise superimposed. This poses significant challenges to signal detection. While optimal maximum-likelihood (ML) detection yields the best performance, its complexity increases exponentially with the number of transmit antennas, making it completely impractical for mMIMO systems. Linear detectors such as Zero Forcing (ZF) and Minimum Mean Square Error (MMSE) offer lower complexity and are easy to implement in practical systems; however, these algorithms exhibit unsatisfactory performance [4]. Nonlinear detection methods, such as Sphere Decoding (SD), Tree Search (TS), and MMSE with Successive Interference Cancellation (MMSE-SIC), can provide better detection accuracy, but their computational complexity grows rapidly with the system dimension, making them impractical for massive MIMO systems.

Recently, artificial intelligence (AI)-based approaches have also been explored for MIMO detection [5,6]. Expectation Propagation (EP) is one such Bayesian-inspired method that has emerged as a promising approximate Bayesian approach [7], which strikes a balance between detection performance and computational complexity in large-scale MIMO detection [8,9]. Regarding the EP algorithm, most existing studies focus on enhancing it by reducing algorithm complexity or improving detection performance [10,11,12,13,14]. Meanwhile, some research efforts have focused on integrating EP with deep learning or neural networks to attain more robust and superior performance [15,16,17,18,19,20].

Although EP has demonstrated excellent performance under ideal independent and identically distributed (i.i.d.) Rayleigh fading channels, massive real-world MIMO systems often experience spatial channel correlation [21,22,23], which poses a significant challenge to EP detection accuracy. There are numerous application scenarios where both the transmitter and receiver are equipped with large-scale antennas with correlation between antennas. Examples include backhaul links [24], millimeter-wave communications [25], V2X communications [26], and communications between UAVs and the ground [27]. With the increase in frequency bands and technological development, future mobile terminals and IoT terminals may also be equipped with large-scale antennas. In addition, for reconfigurable intelligent surface (RIS)-aided communication scenarios, the total equivalent channel from the transmitter to the receiver via the RIS may also exhibit spatial correlation [28]. At the same time, high-dimensional signal detection requires EP or similar Bayesian algorithms, which are quite sensitive to antenna correlation [29].

To address this problem, some existing works have investigated the performance of EP in correlated channels. For instance, [19] and [20] leverage deep learning to optimize EP parameters, while [13] proposes a preconditioned conjugate gradient (pCG) method to replace matrix inversion, thereby improving numerical stability and reducing complexity. Although these approaches demonstrate enhanced robustness under spatial correlation, they generally treat correlation as a secondary evaluation scenario rather than the main design objective.

This limitation becomes even more critical in the context of millimeter-wave (mmWave) massive MIMO—a key enabling technology for 5G and 6G networks. In practical deployments, antennas are packed in close proximity and thus inherently correlated [30], which significantly undermines detection performance. While having full channel state information (CSI) at the transmitter could, in principle, allow techniques such as beamforming to mitigate correlation effects [31], this is difficult to realize in practice: feedback overhead scales prohibitively with the array size, and mmWave channels are highly time-varying, especially in high-mobility scenarios such as communications between cars and base stations.

Motivated by these challenges, in this work, we focus on the effect of transmit antenna correlation and propose a simple precoding scheme to alleviate such correlation and improve EP detection performance in mMIMO systems. Since the eigenvector matrix of the channel transmit correlation matrix asymptotically approaches a discrete Fourier transform (DFT) matrix [22,32], a DFT precoder is effective in eliminating transmit antenna correlation for mMIMO systems. Simulation results show that when the number of antennas at the transmitter and receiver is equal and the transmitted symbols adopt high-order modulation, the proposed DFT precoder can significantly speed up EP convergence and reduce the detection error rate. One particular advantage of the proposed scheme is that it does not require any CSI at the transmitter—neither the instantaneous CSI nor the statistical CSI [33]. To the best of our knowledge, there is no similar method in the existing literature. The proposed precoding can be implemented in the digital domain, as well as in the analog domain [34] or the beam domain [35], since spatial DFT transforms the signal into the beam domain.

*Notations*: Boldface lowercase letters x and uppercase letters X denote column vectors and matrices, respectively. For a matrix **X**, ${\left[X\right]}_{i,j}$ denotes the element in the *i*-th row and *j*-th column. ∥X∥ and ${∥X∥}_{F}$ denotes the 2-norm and the Frobenius norm, respectively. $X^{T}$ and $X^{H}$ denote, respectively, the transpose and the conjugate transpose of X. diag(X) and diag(x), respectively, denote the diagonal extraction operator and the expansion from vector to diagonal matrix, respectively. sort(x) denotes sorting the elements of x in descending order. In addition, E[·] denotes the expectation, and CN(0,A) denotes the circularly symmetric complex Gaussian random vector with covariance matrix A. $\mathrm{CN}\left(x:\mu ,A\right)$ represents the joint probability density function of x, which is circularly symmetric about μ and follows a complex Gaussian distribution with covariance matrix A. Furthermore, the upright j denotes the square root of minus one ($j=\sqrt{-1}$), while the italic *j* denotes an integer. A∝B stands for *A* is proportional to *B*.

## 2. System Model

**Figure 1:**
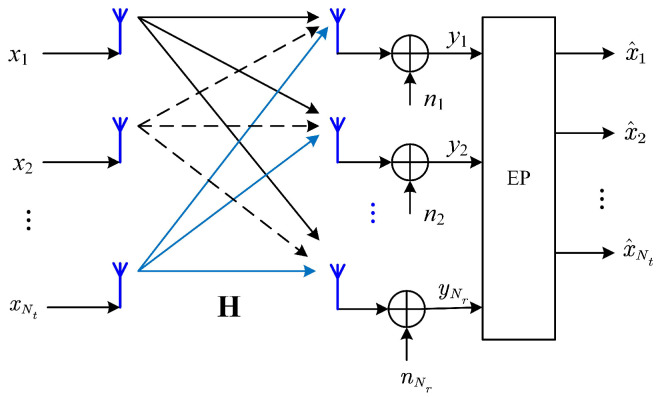
System model.

The system we are considering is a point-to-point narrowband mMIMO system as shown in Figure 1, where the transmitter and the receiver are equipped with $N_{t}$ and $N_{r}$ antennas, respectively. The data symbol vector $x={\left(x_{1},x_{2},\cdots ,x_{N_{t}}\right)}^{T}$ is transmitted over $N_{t}$ transmit antennas. The elements of x are independently and equally likely drawn from the *M*-ary quadrature amplitude modulation (M-QAM) constellation Θ, which is normalized such that $E[|x_{i}{|}^{2}]=1$ for $i=1,2,\cdots ,N_{t}$.

We assume that the time duration of x (i.e., the symbol interval) is much shorter than the channel coherence time, such that the channel does not change during the transmission of x. Then, the received signal can be expressed as

(1) y=Hx+n

where $y={\left(y_{1},y_{2},\cdots ,y_{N_{r}}\right)}^{T}\in C^{N_{r}\times 1}$, $H\in C^{N_{r}\times N_{t}}$ is the mMIMO channel coefficient matrix, and $n∼\mathrm{CN}(0,\sigma_{n}^{2}I)$ is the additive white Gaussian noise (AWGN). The signal-to-noise ratio (SNR) of the system can be defined as $\mathrm{SNR}=N_{t}/\sigma_{n}^{2}$, which is the ratio of the total transmit power ${E[∥x∥}^{2}]=N_{t}$ to the noise power at each antenna.

Similar to most research (e.g., [8]), we also assume that perfect CSI, i.e.,H, is available at the receiver but unknown to the transmitter.

If we ignore the receiver side correlation, the spatial correlation among transmit antennas can be captured as $H=WR^{H/2}$, where W has i.i.d. entries drawn from CN(0,1) [36]. The spatial correlation matrix at the transmitter is given by

(2) $$R=\frac{1}{N_{r}}E\left[H^{H}H\right]=R^{1/2}R^{H/2}.$$

Since the matrix R defined above is Hermitian, it can be decomposed into

(3) $$R=\mathrm{UE}U^{H}$$

where U is a unitary matrix and E is a diagonal matrix whose diagonal elements are the eigenvalues of R.

There exist many models for the correlation matrix R. For example, the geometrical one-ring scattering model [37] assumes no line-of-sight path between the transmitter and receiver, with the departure angle (AoD) of all scatterers relative to the transmitter uniformly distributed around the center AoD θ and an angular spread of Δ. If the transmit antennas are linearly and uniformly distributed at intervals of half a wavelength, then the (i,j)-th elements of R can be computed as

(4) $${\left[R\right]}_{i,j}=\frac{1}{2\Delta }\int_{-\Delta }^{\Delta }e^{-j\pi \left(\alpha +\theta \right)\left(i-j\right)\sin\alpha }d\alpha$$

The most popular model for R in the literature is the exponential model [38], where R is a Toeplitz matrix and its (i,j)-th element is given by

(5) $${\left[R\right]}_{i,j}=\rho^{\left|i-j\right|}$$

where ρ∈[0,1) denotes the correlation coefficient between adjacent antennas. In the following, we will mainly consider Equation (5) as the transmit antenna correlation model.

## 3. Expectation Propagation

### 3.1. Expectation Propagation Algorithm

Expectation Propagation [7] is an approximate Bayesian inference method that seeks a tractable distribution q(x) to approximate the posterior probability distribution p(x|y)∝p(y|x)p(x). In the context of MIMO signal detection problem, $p\left(y|x\right)=\mathrm{CN}\left(y:\mathrm{Hx},\sigma_{n}^{2}I\right)$ is the channel likelihood function, and q(x) is chosen as a complex Gaussian distribution CN(x:μ,Σ) with mean vector μ and covariance matrix Σ. The prior distribution of x, p(x), is approximated as a complex Gaussian distribution $\mathrm{CN}\left(x:\mathrm{diag}{\left(\Lambda \right)}^{-1}\gamma ,\mathrm{diag}{\left(\Lambda \right)}^{-1}\right)$ where $\gamma ={\left(\gamma_{1},\gamma_{2},\cdots ,\gamma_{N_{t}}\right)}^{T}\in C^{N_{t}\times 1}$ and $\Lambda ={\left(\lambda_{1},\lambda_{2},\cdots ,\lambda_{N_{t}}\right)}^{T}\in C^{N_{t}\times 1}$ are variational parameters updated iteratively.

Let *L* be the maximum number of iterations. The EP algorithm proceeds as follows:

(1) Initialization: Set $\lambda_{i}^{\left(1\right)}=1$ and $\gamma_{i}^{\left(1\right)}=0$ for all $i=1,2,\ldots ,N_{t}$. Form vectors $\Lambda^{\left(1\right)}={\left[\lambda_{1}^{\left(1\right)},\lambda_{2}^{\left(1\right)},\ldots ,\lambda_{N_{t}}^{\left(1\right)}\right]}^{T}$ and $\gamma^{\left(1\right)}={\left[\gamma_{1}^{\left(1\right)},\gamma_{2}^{\left(1\right)},\ldots ,\gamma_{N_{t}}^{\left(1\right)}\right]}^{T}$.
(2) Posterior update: At iteration *l*, compute
  (6) $$\begin{array}{cc} \Sigma^{\left(l\right)} & ={\left(\sigma_{n}^{-2}H^{H}H+\mathrm{diag}\left(\Lambda^{\left(l\right)}\right)\right)}^{-1}, \end{array}$$
  (7) $$\begin{array}{cc} \mu^{\left(l\right)} & =\Sigma^{\left(l\right)}\left(\sigma_{n}^{-2}H^{H}y+\gamma^{\left(l\right)}\right). \end{array}$$
(3) Cavity distribution: For each symbol $x_{i}$, compute the parameters of cavity distribution $q_{\setminus i}\left(x_{i}\right)=\mathrm{CN}\left(x_{i}:t_{i}^{\left(l\right)},h_{i}^{2(l)}\right)$ as
  (8) $$\begin{array}{cc} h_{i}^{2(l)} & =\frac{\Sigma_{ii}^{\left(l\right)}}{1-\Sigma_{ii}^{\left(l\right)}\lambda_{i}^{\left(l\right)}}, \end{array}$$
  (9) $$\begin{array}{cc} t_{i}^{\left(l\right)} & =h_{i}^{2(l)}\left(\frac{\mu_{i}^{\left(l\right)}}{\Sigma_{ii}^{\left(l\right)}}-\gamma_{i}^{\left(l\right)}\right). \end{array}$$
(4) Moment matching and parameters update: Define the distribution $\tilde{p}\left(x_{i}\right)\propto q_{\setminus i}\left(x_{i}\right)p\left(x_{i}\right)$, compute its mean and variance:

(10) $$\begin{array}{cc} m_{i}^{\left(l\right)} & =\frac{\sum_{x_{i}\in \Theta }x_{i}\exp\;\left(-\frac{|x_{i}-t_{i}^{\left(l\right)}{|}^{2}}{h_{i}^{2(l)}}\right)}{\sum_{x_{i}\in \Theta }\exp\;\left(-\frac{|x_{i}-t_{i}^{\left(l\right)}{|}^{2}}{h_{i}^{2(l)}}\right)}, \end{array}$$

(11) $$\begin{array}{cc} V_{i}^{\left(l\right)} & =\frac{\sum_{x_{i}\in \Theta }{\left|x_{i}-m_{i}^{\left(l\right)}\right|}^{2}\exp\;\left(-\frac{|x_{i}-t_{i}^{\left(l\right)}{|}^{2}}{h_{i}^{2(l)}}\right)}{\sum_{x_{i}\in \Theta }\exp\;\left(-\frac{|x_{i}-t_{i}^{\left(l\right)}{|}^{2}}{h_{i}^{2(l)}}\right)}, \end{array}$$

where Θ is the modulation constellation set. Next, update the variational parameters as

(12) $$\begin{array}{cc} \lambda_{i}^{\left(l+1\right)} & =\frac{1}{V_{i}^{\left(l\right)}}-\frac{1}{h_{i}^{2(l)}}, \end{array}$$

(13) $$\begin{array}{cc} \gamma_{i}^{\left(l+1\right)} & =\frac{m_{i}^{\left(l\right)}}{V_{i}^{\left(l\right)}}-\frac{t_{i}^{\left(l\right)}}{h_{i}^{2(l)}}. \end{array}$$

To stabilize convergence, apply damping with factor β∈(0,1]:

(14) $$\begin{array}{cc} \lambda_{i}^{\left(l+1\right)} & \leftarrow \left(1-\beta \right)\lambda_{i}^{\left(l\right)}+\beta \lambda_{i}^{\left(l+1\right)}, \end{array}$$

(15) $$\begin{array}{cc} \gamma_{i}^{\left(l+1\right)} & \leftarrow \left(1-\beta \right)\gamma_{i}^{\left(l\right)}+\beta \gamma_{i}^{\left(l+1\right)}. \end{array}$$

(5) Iteration: Repeat steps 2–4 until convergence or the maximum number of iterations *L* is reached.
(6) Decision: The final detection output is taken as

(16) $$\hat{x}=\mathrm{Hard}\left(\mu^{\left(L\right)}\right),$$

where the hard-decision function Hard(·) maps the elements of $\mu^{\left(L\right)}$ to a point in Θ.

In summary, the EP algorithm constructs a Gaussian approximation to the posterior distribution of the transmitted signal. During the iterative process, the discrepancy between the first- and second-order moments of the symbol-wise posterior distribution and its Gaussian approximation is progressively reduced. For high-dimensional signal detection with high-order modulation, the EP algorithm converges rapidly and achieves significant performance gains over conventional methods [8].

### 3.2. Transmit Antenna Correlation Degrades the Performance of EP

**Figure 2:**
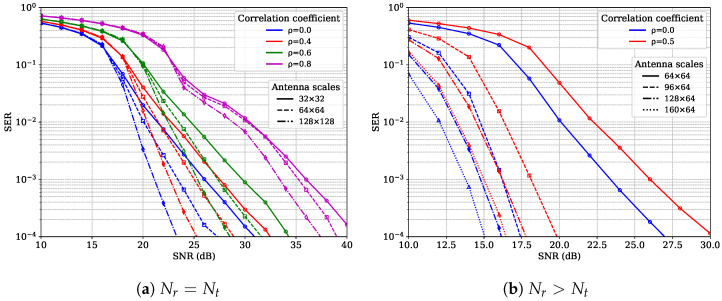
SER performance of EP versus SNR under different correlated channels with 16-QAM modulation.

The performance of the EP algorithm is closely related to the characteristics of the channel matrix H. One of the unfavorable factors in the channel structure is the correlation of transmit antennas [29,39]. We have conducted extensive simulations, and the results show that the correlation between transmit antennas degrades the performance of the EP algorithm. The higher the correlation coefficient, the closer the number of transmit and receive antennas, and the higher the modulation order, the more severe the performance loss. Figure 2 shows an example of the simulation results. Figure 2a shows the symbol error rate (SER) performance of a MIMO system with different numbers of antennas under different channel correlations, where the number of transmit and receive antennas is configured to be the same. As the antenna correlation increases (i.e., larger ρ), SER performance degrades significantly. Compared with the uncorrelated i.i.d. Rayleigh channel (ρ=0), the performance loss caused by high correlation is quite significant. When ρ=0.8, the performance loss at SER = $1\times 10^{-3}$ is about 10.1 dB, 12.1 dB and 13.1 dB for $N_{t}=32,64$, and 128, respectively. Figure 2b shows the SER performance of a MIMO system with different numbers of receive antennas, while the number of transmit antennas is fixed at $N_{t}=64$. As the number of receive antennas increases, the SER performance improves significantly, and the impact of transmit antenna correlation diminishes accordingly. Therefore, the scenario where the impact of transmit antenna correlation most needs to be addressed is when the number of transmit antennas is equal to the number of receive antennas.

The impact of transmit antenna correlation on the performance of EP detection is understandable. Consider a MIMO system where only two symbols $x_{1},x_{2}$ are transmitted over antenna 1 and antenna 2, respectively. The received signal is given by

(17) $$y=h_{1}x_{1}+h_{2}x_{2}+n,$$

where $h_{1}$ and $h_{2}$ are the first two columns of H. Given the received signal y, the posterior distribution of $x={\left[x_{1}\;x_{2}\right]}^{T}$ is

(18) $$p\left(x|y\right)\;\propto \;\exp\;\left(-\frac{1}{\sigma_{n}^{2}}\;∥y-h_{1}x_{1}-h_{2}x_{2}{∥}^{2}\right).$$

Using a complete orthonormal basis $v_{1},v_{2}$, $h_{1}$ and $h_{2}$ can be decomposed as $h_{1}=∥h_{1}∥v_{1}$ and $h_{2}=\alpha_{1}v_{1}+\alpha_{2}v_{2}$, where $v_{2}⊥v_{1}$, $∥v_{1}∥=∥v_{2}∥=1$. The projection of y onto the space spanned by $\left\{v_{1},v_{2}\right\}$ is $\tilde{y}=\beta_{1}v_{1}+\beta_{2}v_{2}$. Since $\tilde{y}$ is the sufficient statistics for the signal detection, by replacing y with y and substituting the orthogonal expansion of $h_{1}$ and $x_{2}$ into (18), we have

(19) $$p\left(x_{1},x_{2}|y\right)\propto \exp\;\left(-\frac{1}{\sigma_{n}^{2}}|\beta_{1}-∥h_{1}∥\;x_{1}-\alpha_{1}x_{2}{|}^{2}\right)\;\exp\;\left(-\frac{1}{\sigma_{n}^{2}}|\beta_{2}-\alpha_{2}x_{2}{|}^{2}\right).$$

This indicates that $x_{1}$ and $x_{2}$ are posteriorly correlated. The EP algorithm tries to solve the posterior mean by iteratively approximating x as independent Gaussian variables. The posterior correlation shown in (19) will affect the Gaussian approximation in the EP algorithm, thereby influencing the convergence speed and convergence accuracy of the EP algorithm. Specifically, from the principle and procedure of the EP algorithm, it can be seen that the posterior distribution is approximated by a joint Gaussian distribution with mean μ and covariance Σ. However, after updating μ and Σ, the cavity distribution is computed using only the diagonal elements of Σ. Subsequently, moment matching and parameter updates are carried out independently for each symbol. As a result, spatial channel correlation induces posterior dependence among symbols, which conflicts with the independence assumption inherent in EP, thereby leading to performance degradation.

Note that the correlation coefficient of $x_{1}$ and $x_{2}$ conditioned on a given signal y is dictated by the magnitude of $\alpha_{1}=v_{1}^{H}h_{2}={∥h_{1}∥}^{-1}h_{1}^{H}h_{2}$. With two highly correlated transmit antennas, we have a larger $\rho =\frac{1}{N_{r}}E\left[h_{1}^{H}h_{2}\right]$, which implies a lower probability of $\alpha_{1}$ being small, a higher chance of $x_{1}$ and $x_{2}$ being highly correlated, and hence a higher approximation error in EP.

The impact of antenna correlation can also be observed through the cavity variance, which is defined in (8). A larger cavity variance $h_{i}^{2}$ indicates lower symbol reliability, and consequently, a higher detection error probability [8,39].

**Figure 3:**
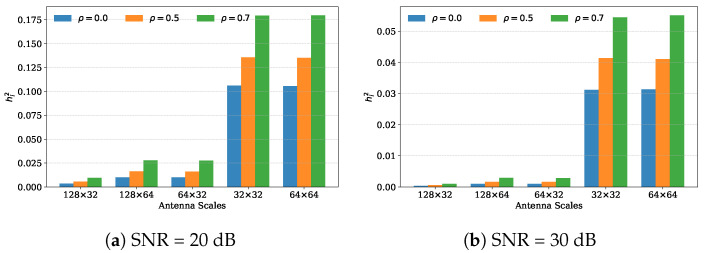
$h^{2}$’s mean versus antenna scales and ρ.

At the initialization stage, following [10], we set γ=0 and $\Lambda_{i}=\lambda =1$. By decomposing $H=USV^{H}$ and combining Equations (6)–(8), the initial cavity variance for symbol $x_{i}$ is given by

(20) $$h_{i}^{2}=\frac{\sum_{j=1}^{N_{t}}(\frac{\sigma_{n}^{2}}{S_{j}^{2}+\lambda \sigma_{n}^{2}}|v_{ij}{|}^{2})}{\sum_{j=1}^{N_{t}}(\frac{S_{j}^{2}}{S_{j}^{2}+\lambda \sigma_{n}^{2}}|v_{ij}{|}^{2})},$$

where $v_{ij}$ is the (i,j)-th element of the right singular matrix V, and $S_{j}$ is the *j*-th singular value. Equation (20) indicates that the singular values S and the energy distribution in V have a great influence on cavity variance $h^{2}$. Figure 3 shows some statistical results of the cavity variance obtained via Monte Carlo simulations. We can see that the initial $h^{2}$ for correlated channels is uniformly larger than that in the i.i.d. case. This is especially evident when the transmitter and receiver have the same number of antennas ($N_{t}=N_{r}$). If the receiver is equipped with more antennas than the transmitter ($N_{r}>N_{t}$), $h^{2}$ is significantly smaller. This enables us to predict that when the number of receive antennas is greater than that of transmit antennas, the performance of EP improves rapidly, and the impact of antenna correlation on it weakens accordingly, which is consistent with the observations in Figure 2.

## 4. Eliminating Antenna Correlation Using DFT Precoding

In MIMO systems, precoding/beamforming generally stands for a linear mapping that transforms the original data symbols into a vector to be transmitted over multiple antennas [31,40,41]. Theoretically, the optimal mapping should be channel-specific—the precoder should be optimized with specific H.

In large-scale antenna systems, it is no easy task for the transmitter to obtain sufficiently accurate CSI, which often results in a certain overhead burden. As wavelengths become increasingly shorter, even statistical CSI is not easy to obtain accurately. For instance, in millimeter-wave communications, the sub-centimeter-level movements of surrounding pedestrians and vehicles can alter the propagation environment and affect the channel’s correlation matrix. In this paper, we focus on precoding schemes that do not require instantaneous CSI, nor do they depend on the specific channel correlation matrix. This is possible because in mMIMO systems, the correlation matrices share some common structural features. For example, the correlation coefficient decreases rapidly as the antenna spacing increases, meaning the entire correlation matrix is mainly determined by the correlation between adjacent antennas.

With a precoding matrix (precoder) $P\in C^{N_{t}\times N_{t}}$ applied in the system, the original signal vector x is first transformed into Px, and then transmitted through $N_{t}$ transmit antennas. We assume that precoding does not change the average transmit power of the signal, i.e., ${E[∥\mathrm{Px}∥}^{2}]=E\left[∥x{∥}^{2}\right]$. The received signal becomes

(21) $$y\;=\;HPx+n\;=\;\tilde{H}x+n,$$

where $\tilde{H}=HP$ is the equivalent channel matrix taking into account the effect of precoding.

From the perspective of the EP algorithm, $\tilde{H}$ is the channel matrix input for executing the algorithm. Similar to Equation (2), the equivalent transmitter-side correlation matrix for this equivalent channel matrix is given by

(22) $$R_{\mathrm{eq}}=\frac{1}{N_{r}}E\left[P^{H}H^{H}\mathrm{HP}\right]=P^{H}RP=P^{H}{\mathrm{UEU}}^{H}P.$$

Now, it is obvious that by choosing the precoding matrix as P=U, the equivalent correlation matrix becomes $R_{\mathrm{eq}}=E$, where all the off-diagonal elements are zero, implying that the transmit antenna correlation has been completely eliminated.

For uniform linear arrays, the correlation matrix has a Toeplitz form [22]. By using Szegö’s theorem [32], R will approach a circulant matrix for sufficiently large $N_{t}$. It is well known that the eigenvector matrix of a circulant matrix is the unitary DFT matrix. So we have

(23) $$R\approx F\tilde{E}F^{H},$$

where F is the normalized DFT matrix, $\tilde{E}$ is a diagonal matrix, which is actually E with its diagonal elements permuted.

Equation (23) is equivalent to $F^{H}\mathrm{RF}\approx \tilde{E}$. The approximation error can be measured by comparing the diagonal elements of $F^{H}\mathrm{RF}$ with those of $\tilde{E}$. Define

(24) $$e=\frac{{∥\mathrm{sort}\left(\mathrm{diag}(F^{H}\mathrm{RF})\right)-\mathrm{sort}\left(\mathrm{diag}(E)\right)∥}^{2}}{{∥E∥}_{F}^{2}}.$$

**Figure 4:**
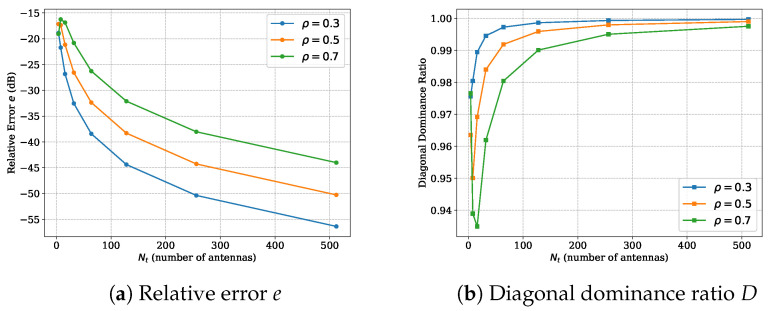
Comparison of the proximity between DFT precoder and EVD precoder.

It is obvious that the smaller the value of *e*, the closer $F^{H}\mathrm{RF}$ is to a diagonal matrix, and the closer F approaches U (up to column permutation). Figure 4a is the simulation results of *e* under different correlation levels. As $N_{t}$ increases, *e* decreases, confirming that in the large-scale regime, the DFT matrix is very close to the eigenvector matrix of R.

Another method to evaluate the approximation error is to observe the diagonal property of $R_{\mathrm{eq}}=P^{H}\mathrm{RP}$. If F=U, then $R_{\mathrm{eq}}=E$ must be a diagonal matrix. The deviation between F and U will result in non-zero off-diagonal elements of $R_{\mathrm{eq}}$. Define diagonal dominance ratio of $R_{\mathrm{eq}}$ as

(25) $$D=\frac{∥\mathrm{diag}\left(R_{\mathrm{eq}}\right){∥}^{2}}{∥R_{\mathrm{eq}}{∥}_{F}^{2}}.$$

The degree to which *D* approaches 1 represents the degree to which F approaches U. We can clearly see from Figure 4b that as the number of antennas increases, *D* will become very close to 1.

A conclusion we can immediately draw from the above analysis is that for massive MIMO systems, if we choose the precoding matrix P=F, then we will have $R_{\mathrm{eq}}\approx \tilde{E}$; that is, the adoption of DFT precoding can effectively eliminate the correlation among transmit antennas.

## 5. Simulation Results

In this section, we use simulations to verify our proposed precoding scheme. We select no precoding and eigenvalue decomposition (EVD)-based precoding (i.e., P=U) as the baselines. In all simulations, we adopt Equation (5) as the model for the correlation matrix, set L=5 as the number of iterations for the EP algorithm, and use β=0.5 as the damping factor in Equations (14) and (15).

**Figure 5:**
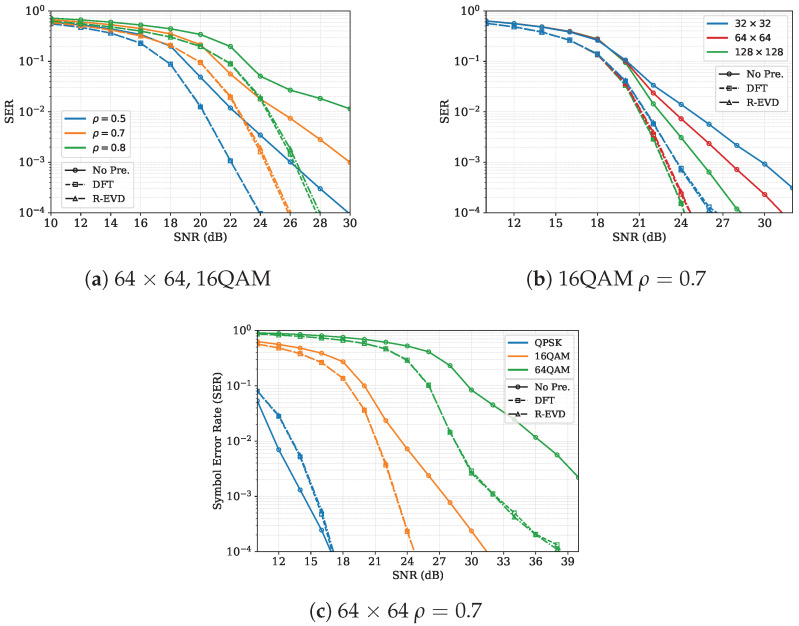
SER performance of EP detection with various precoding schemes under transmit-side correlation.

Simulation results for a 64×64 MIMO system with 16-QAM under transmit-side correlation are summarized in Figure 5a. It can be observed that the performance of the proposed DFT precoding matches that of the EVD-based precoding scheme very well. At an SER of $1\times 10^{-3}$, the performance gains are about 4 dB, 5.2 dB, and 8 dB for transmit correlation coefficients ρ=0.5, ρ=0.7, and ρ=0.8, respectively. Figure 5b examines the proposed precoding scheme under varying antenna configurations. At $\mathrm{SER}=10^{-3}$, the gains achieved via the proposed DFT precoding for the 32×32, 64×64, and 128×128 antenna configurations are approximately 6.2 dB, 5 dB, and 4 dB, respectively. Figure 5c presents the simulation results for different modulation orders, including quadrature phase shift keying (QPSK), 16-QAM, and 64-QAM. At $\mathrm{SER}=10^{-3}$, the SNR gains relative to the unprecoded scheme are approximately 5 dB for 16-QAM and 9 dB for 64-QAM, while a slight performance reduction is observed for QPSK. This is because QPSK is less affected by spatial correlation. This result indicates that higher-order modulations derive greater benefits from precoding under correlated channel conditions.

The proposed DFT precoding scheme only modifies the transmitter without altering the EP algorithm at the receiver; thus, it does not affect the receiver’s algorithm complexity or hardware cost. Introducing precoding at the transmitter does introduce a certain degree of computational or hardware complexity if DFT is implemented in the analog domain or via lens [35]. However, due to the availability of fast DFT algorithms, the increase in complexity is limited. If this scheme is applied to a wideband MIMO-OFDM system, the complexity of precoding will be comparable to that of OFDM modulation.

## 6. Conclusions

As the number of antennas continues to grow and transmit antennas are deployed with increasing density, coupling and correlation between antennas become increasingly unavoidable. On the other hand, the signal dimensionality brought by large-scale antennas makes it difficult for traditional signal detection methods to achieve good performance, leaving Bayesian methods such as EP as the only viable option. However, antenna correlation has a significant impact on the performance of the EP algorithm. This paper proposes a simple CSI-free scheme that can effectively eliminate the correlation between antennas and significantly improve system performance by implementing the DFT transform at the transmitter.

Although DFT precoding can mitigate performance losses caused by antenna correlation, and some qualitative discussions have been provided in this paper, how the structural characteristics of the channel matrix affect the EP algorithm remains an important issue to be addressed.

## Data Availability

The original contributions presented in this study are included in the article. Further inquiries can be directed to the corresponding author.
